# IgG4-related disease presenting as central skull base osteomyelitis with deep neck abscesses: A case report

**DOI:** 10.1097/MD.0000000000043351

**Published:** 2025-07-18

**Authors:** Shen-Han Lee, Rohaizam Jaafar, Nurul Akmar Misron, Zulkifli Yusof, Nik Adilah Nik Othman

**Affiliations:** a Department of Otorhinolaryngology-Head & Neck Surgery, School of Medical Sciences, Universiti Sains Malaysia, Health Campus, Kubang Kerian, Kelantan, Malaysia; b Hospital Pakar Universiti Sains Malaysia, Universiti Sains Malaysia, Health Campus, Kubang Kerian, Kelantan, Malaysia; c Department of Otorhinolaryngology, Hospital Sultanah Bahiyah, Ministry of Health Malaysia, Alor Setar, Kedah, Malaysia; d Department of Pathology, Hospital Sultanah Bahiyah, Ministry of Health Malaysia, Alor Setar, Kedah, Malaysia.

**Keywords:** case report, deep neck abscess, IgG4-related disease, immunoglobulin G4, osteomyelitis, skull base

## Abstract

**Rationale:**

IgG4-related disease (IgG4-RD) is an immune-mediated, systemic chronic inflammatory condition that can arise in the head and neck as a tumor-like mass. We report the first case of IgG4-RD manifesting as central skull base osteomyelitis with deep neck abscesses.

**Patient concerns:**

A 68-year-old Malay female with diabetes presented with a 2-month history of worsening parietal headache radiating to the neck, intermittent fever, and left-sided tinnitus.

**Diagnoses:**

Otoscopic and cranial nerve examinations were normal, while nasoendoscopy revealed an erythematous nasopharynx with slight bilateral obliteration of the Fossa of Rosenmüller; however, biopsies showed no malignancy. Blood investigations revealed hyperglycemia and elevated inflammatory markers, while imaging revealed central skull base osteomyelitis with chronic retropharyngeal and parapharyngeal abscesses. Despite initial symptomatic improvement with intravenous ceftriaxone and glycemic control, our patient presented a month later with left facial nerve palsy (House–Brackmann Grade IV), worsening bilateral hearing loss (right moderate-to-severe mixed hearing loss and left moderate-to-profound mixed hearing loss), and hyperglycemia. A deep biopsy of the retropharyngeal lesion under general anesthesia revealed a diagnosis of IgG4-RD.

**Interventions:**

Treatment with oral prednisolone resulted in symptomatic improvement and resolution of the deep neck abscesses. Azathioprine was started after steroid was tapered over 2 months but discontinued due to the patient developing headache.

**Outcomes:**

The patient made good symptomatic recovery although she developed right sigmoid sinus thrombosis at 5 months follow-up, for which she was started on warfarin. At 18 months follow-up, she remained well with slight facial weakness (House–Brackmann Grade II) and improved hearing (mild-to-moderate bilateral sensorineural hearing loss).

**Lessons:**

Our case highlights central skull base osteomyelitis with deep neck abscesses as a new clinical manifestation of IgG4-RD. IgG4-RD may not always present as a tumor-like mass de novo but instead presents with features of an infection in immunosuppressed individuals. In patients with treatment-refractory skull base osteomyelitis and deep neck abscesses, IgG4-RD should be considered as a differential diagnosis and a tissue biopsy is warranted.

## 1. Introduction

IgG4-related disease (IgG4-RD) is a multisystem chronic fibroinflammatory condition that can affect any organ where it typically presents as tumor-like masses with hallmark histopathological features.^[1]^ After the pancreatobiliary system, the head and neck region is the second most common anatomical site affected by IgG4-RD. The 2019 classification criteria for IgG4-RD developed by the American College of Rheumatology/European Alliance of Associations for Rheumatology (formerly European League Against Rheumatism) (ACR/EULAR) identified major head and neck sites involved by IgG4-RD as the salivary glands, orbit, lacrimal glands, thyroid, and meninges.^[2]^ The skull base is less commonly involved but IgG4-RD may present in this region as a tumor-like mass mimicking nasopharyngeal carcinoma, pituitary tumor, or meningioma.^[3]^ Very rarely, IgG4-RD has been reported to involve lateral skull base structures such as the temporal bone and middle ear with clinical and radiological features mimicking skull base osteomyelitis.^[4]^

Skull base osteomyelitis is a rare, potentially life-threatening condition that occurs as a complication of otologic or sinus infection classically seen in elderly, diabetic or immunocompromised patients.^[5]^ Two forms of skull base osteomyelitis exist—typical and atypical skull base osteomyelitis. Typical skull base osteomyelitis affects the temporal bone and occurs as a result of necrotizing otitis externa from *Pseudomonas* species infection.^[5,6]^ In contrast, atypical or central skull base osteomyelitis occurs without a preceding ear infection and involves the basiphenoid and basiocciput via spread of infections from the paranasal sinuses, deep face or oral cavity.^[5,7]^ It is relatively easier to diagnose typical skull base osteomyelitis owing to its classical presentation whereas central skull base osteomyelitis is often difficult to diagnose due to its nonspecific initial presentation. For any infiltrative or destructive process of the central skull base, malignancies such as nasopharyngeal carcinoma or lymphoma need to be ruled out via skull base or nasopharyngeal biopsies.^[7]^ Rare differential diagnoses considerations include nonneoplastic diseases such as granulomatosis with polyangiitis, tuberculosis, sarcoidosis, fibrous dysplasia and Paget’s disease.^[7]^

Here, we report the first case of a patient with IgG4-RD that initially manifested as central skull base osteomyelitis with deep neck abscesses that subsequently organized into a tumor-like mass after several months.

## 2. Case presentation

A 68-year-old Malay female presented with a 2-month history of worsening parietal headache radiating down the neck, intermittent fever, and left-sided tinnitus. Her past medical history was significant for type II diabetes mellitus although there was no significant past surgical, family or psychosocial history. She was previously a housewife, widowed for over 10 years, and lives with her eldest son and his family. She did not smoke or drink alcohol.

**Figure 1.**
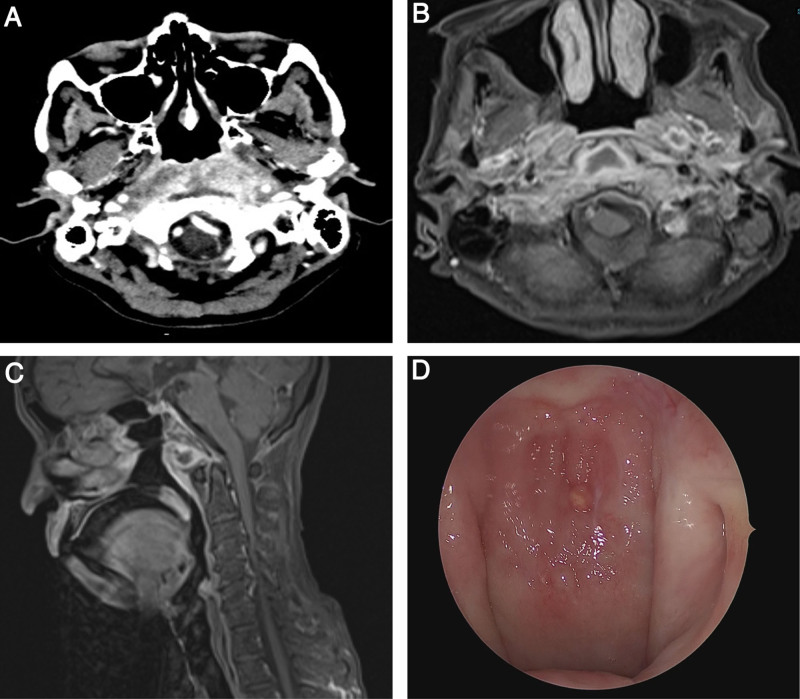
(A) Axial CT scan showing heterogeneous retropharyngeal soft tissue enhancement at the level of the skull base and nasopharynx and a small abscess (0.7 × 2.3 cm) anterior to the clivus with bony erosion. (B) Post-contrast T2-weighted axial MRI scan showing multiloculated collections in the retropharyngeal (1.2 × 1.6 × 1.4 cm) and left parapharyngeal (0.8 × 1.0 × 0.9 cm) regions with peripheral enhancement, suggestive of abscesses. (C) Post-contrast T2-weighted sagittal MRI scan showing the retropharyngeal abscess and clival osteomyelitis. (D) Endoscopic view of the midline nasopharyngeal mass. CT = computed tomography.

The otoscopic and cranial nerve examinations were normal. Nasoendoscopy showed an erythematous nasopharynx and slight obliteration of the Fossa of Rosenmüller bilaterally; however, nasopharyngeal mucosal biopsies were negative for malignancy. Laboratory investigations revealed elevated white cells (16.78 cells × 10^9^/L), C-reactive protein (92.07 mg/L), erythrocyte sedimentation rate (94.0 mm/h), glucose (19.1 mmol/L), and glycated hemoglobin (6.9%). Pure tone audiometry revealed mild high-frequency sensorineural hearing loss bilaterally. Computed tomography revealed heterogeneous enhancement of the retropharyngeal soft tissue at the skull base and nasopharynx, indicating extensive inflammation as well as clival osteomyelitis with a small abscess (Fig. 1A). With an initial working diagnosis of skull base osteomyelitis, she was treated with intravenous ceftriaxone as empirical coverage for 2 weeks with initial symptomatic improvement and switched to a basal-bolus insulin regimen for hyperglycemia (capillary blood glucose range: 13.5–22.3 mmol/L).

During follow-up 1 month later, she presented with left facial nerve palsy (House–Brackmann Grade IV), bilateral worsening hearing loss, and poor oral intake. Investigations revealed worsening inflammatory markers, hyperglycemia, and hyponatremia (121 mmol/L) due to dehydration from poor oral intake. The patient was readmitted for supportive care and treatment with intravenous ceftazidime, another third-generation cephalosporin with better activity against *Pseudomonas aeruginosa* (*P aeruginosa*), a common pathogen in skull base osteomyelitis. Pure tone audiometry revealed right moderate-to-severe mixed hearing loss and left moderate-to-profound mixed hearing loss. Chest radiography was normal, whereas tuberculosis and melioidosis work-up yielded negative results. Magnetic resonance imaging (MRI) revealed abscesses in the retropharyngeal and left parapharyngeal spaces with extensive inflammation and clival osteomyelitis (Fig. 1B, C).

**Figure 2.**
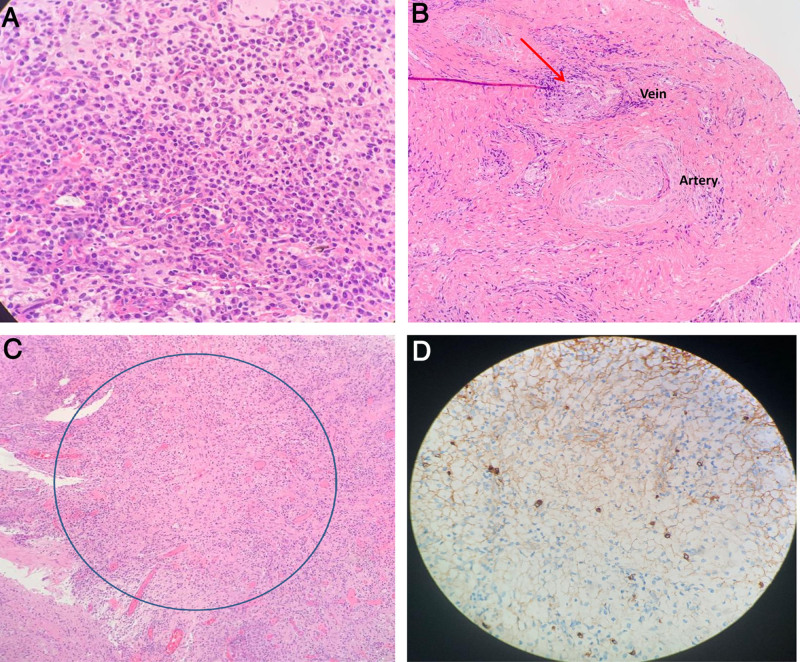
Histopathologic and immunohistochemical examination demonstrated hallmark features of IgG4-RD. (A) Dense lymphoplasmacytic infiltrates on hematoxylin and eosin (H & E) stain (10×). (B) Obliterative phlebitis involving blood vessel (arrow) on H & E stain (4×). (C) Storiform fibrosis on H & E stain (10×). (D) Immunostaining for IgG4 shows on average 5–12 IgG4-positive cells per high-power field (10×). IgG4-RD = immunoglobulin G4-related disease.

As her symptoms did not improve despite 2 weeks of antibiotic treatment, an endoscopic-guided deep biopsy of the retropharyngeal lesion under general anesthesia was performed. Intraoperatively, there was a new midline nasopharyngeal nodular mass (Fig. 1D), and an incision through the mucosa and buccopharyngeal fascia revealed a friable mass in the retropharyngeal space. Biopsies of the mass showed histopathological and immunohistochemical features consistent with IgG4-RD (Fig. 2). The criteria for the diagnosis of IgG4-RD met by the biopsy include dense lymphoplasmacytic infiltrate, storiform-type fibrosis, and obliterative phlebitis. The number of IgG4-positive plasma cells was between 5 and 12 IgG4-positive cells per high-power field, on average. However, the ratio of IgG4/IgG-positive cells were not able to be quantified due to inconclusive IgG staining on the tissue biopsy sample.

She was started on oral prednisolone 40 mg daily for 2 weeks, tapered over 2 months to a maintenance dose of 15 mg daily. Oral azathioprine 50 mg daily was started at the end of the steroid tapering period as a steroid-sparing immunosuppressive agent to maintain remission. However, azathioprine was discontinued when the patient developed headache, and oral prednisolone was continued at a dose of 15 mg daily. Two months after steroid therapy, her headaches resolved, facial weakness improved to House–Brackmann Grade II, and repeat nasoendoscopy showed reduction of the lesion size and edema. Despite this initial improvement, her headaches recurred after 5 months. Contrast-enhanced computed tomography scan showed right sigmoid sinus thrombosis with hydrocephalus, although the deep neck abscesses resolved. She was started on anticoagulation with oral warfarin 4 mg daily bridged with subcutaneous enoxaparin. At 18 months follow-up, she remained well with only slight facial weakness (House–Brackmann Grade II) and improved hearing, with mild-to-moderate bilateral sensorineural hearing loss on audiometry.

## 3. Discussion

To our knowledge, this is the first report of IgG4-RD presenting as central skull base osteomyelitis with deep neck space abscesses. In the largest multi-institutional case series of skull base IgG4-RD reported by Marinelli et al, all patients presented with mass lesions, but none had skull base osteomyelitis or deep neck abscess.^[3]^ The clivus, part of the central skull base, is rarely affected by IgG4-RD, with a systematic review reporting only 11 cases of clival involvement.^[8]^ Of these, 10 cases presented with mass-like lesions in the clivus, whereas only 1 case involved clival osteomyelitis without deep neck space involvement.^[8]^ While highly unusual, the clinical presentation of IgG4-RD as abscesses has been reported in other anatomic sites such as the breast,^[9]^ liver,^[10]^ and suppurative cervical lymph nodes.^[11]^

The management of this case demonstrated several strengths. The timely recognition of skull base osteomyelitis and the use of intravenous antibiotics provided initial symptomatic relief. Regular follow-up and reassessment allowed for detection of treatment failure and subsequent complications such as facial nerve palsy and worsening hearing loss. This also led to the decision to perform a deep, endoscopic-guided biopsy, which was pivotal in clinching the definitive diagnosis of IgG4-RD and a shift to the use of immunosuppressive therapy with corticosteroids. However, key limitations of this case include diagnostic delay due to the atypical clinical presentation, nonspecific imaging findings and the initial negative nasopharyngeal biopsies, which may have contributed to disease progression.

This case posed a diagnostic challenge for several reasons. First, the extension of skull base osteomyelitis into the deep neck space is exceedingly rare, but possible as the skull base forms the superior border of the retropharyngeal space. Second, MRI revealed T1-weighted hypointensity and T2-weighted hyperintensity with peripheral contrast enhancement consistent with abscesses. In contrast, typical IgG4-RD lesions, including those of the skull base, are isointense on T1-and hypointense on T2-weighted MRI, with homogeneous contrast enhancement, indicating increased cellularity and fibrosis.^[3,12]^ The involvement of the facial nerve and worsening hearing at this stage might suggest the spread of osteomyelitis involving the petrous temporal bone, despite not being evident on MRI. The extensive local inflammation may also have contributed to the late sequela of sigmoid sinus thrombosis from retrograde thrombophlebitis via the emissary veins.

The differential diagnoses for this case include infectious skull base osteomyelitis, particularly from bacterial or fungal pathogens such as *P aeruginosa*, *Staphylococcus aureus*, or mucormycosis, in light of the patient’s diabetes, fever, and imaging findings. Tuberculous osteomyelitis or deep neck tuberculosis also need to be considered, especially in tuberculosis-endemic regions such as Malaysia. Malignancies such as nasopharyngeal carcinoma, lymphoma, or metastatic disease must be excluded, particularly with nasoendoscopic findings of Fossa of Rosenmüller obliteration. Granulomatosis with polyangiitis is also a possible differential, as it may present with skull base involvement and cranial neuropathies. Other inflammatory conditions such as sarcoidosis and idiopathic inflammatory pseudotumor could also mimic the presentation. Ultimately, the diagnosis of IgG4-related disease was confirmed by deep biopsy, histopathology and immunostaining. This underscores the importance of considering the diagnosis of IgG4-RD in treatment-refractory skull base osteomyelitis with deep neck abscesses.

A comparison with other skull base IgG4-RD cases in the literature reveal consistent diagnostic challenges due to its mimicry of malignancy and infection, particularly when imaging is nonspecific and serum IgG4 levels are normal. Furthermore, imaging features often overlap with tumors or infection, leading to delayed diagnosis. Several case reports highlight the need for deep tissue biopsy as superficial samples often yield inconclusive results. For instance, Detiger et al described a case series in which IgG4-RD mimicked nasopharyngeal carcinoma or skull base tumors, and deep tissue biopsy was necessary to reach the diagnosis after superficial biopsies were inconclusive.^[13]^ Similarly, Liu et al reported a patient with IgG4-RD involving the skull base that initially appeared clinically and radiologically indistinguishable from nasopharyngeal cancer, and required deep biopsies for histopathological confirmation.^[14]^

The 2019 ACR/EULAR classification criteria for the diagnosis of IgG4-RD was originally developed for use in research settings to identify relatively homogeneous IgG4-RD populations for clinical trials.^[2]^ While highly specific for the diagnosis of IgG4-RD in patients with characteristic organ involvement, it suffers from a lack of sensitivity in identifying rare organ manifestations such as skull base involvement.^[15]^ In contrast, the 2020 revised comprehensive diagnostic criteria for IgG4-RD developed in Japan adopts a more pragmatic and broader approach—it allows for diagnosis based on a combination of clinical, serological, and histopathological findings, and include cases with atypical localization.^[16]^ Our case exemplifies how the revised comprehensive diagnostic criteria are better suited for capturing rare, localized disease presentations in real-world clinical contexts, since the 2019 ACR/EULAR criteria may not accommodate such rare anatomical involvement, especially in the absence of classic organ involvement (e.g., pancreas, salivary glands). Our case illustrates how IgG4-RD can present outside the classical patterns and defy initial diagnostic criteria.

Our observation of an abscess in the incipient stages of pseudotumor formation raises the question of whether immunosuppression due to uncontrolled diabetes influences the IgG4-RD phenotype. Recent studies have suggested that IgG4-RD presents with a range of tissue phenotypes, progressing from a proliferative (inflammatory) to fibrotic phase.^[17]^ This balance may depend on the activities of infiltrating CD4 + and CD8 + T cells and B cells as well as fibroblasts that orchestrate fibrosis.^[18]^ It is plausible that diabetes-related immunosuppression may disrupt this balance, leading to a chronic abscess organized via fibrosis.

The treatment approach in this case was guided by the evolving clinical presentation and the eventual diagnosis of IgG4-RD. Empirical broad-spectrum third generation cephalosporin antibiotics, initially intravenous ceftriaxone and then ceftazidime, were chosen based on the initial working diagnosis of skull base osteomyelitis and deep neck abscess, which are often caused by gram-negative and anaerobic organisms. Once histopathology confirmed the diagnosis of IgG4-RD, systemic corticosteroid therapy was initiated with oral prednisolone, the first-line treatment known to induce remission by suppressing the aberrant immune response. Azathioprine, an immunosuppressive steroid-sparing agent, was introduced as maintenance therapy to minimize long-term steroid exposure and reduce relapse risk, but had to be discontinued due to intolerable side effects experienced by this patient. In the event of disease relapse, alternative immunosuppressive agents may be considered, such as mycophenolate mofetil, methotrexate, or rituximab.

Owing to its rarity, there is no level 1 evidence to guide IgG4-RD treatment. While there are cases of spontaneous remission,^[19]^ an international consensus recommends corticosteroids as the first-line therapy, with other immunomodulators reserved for refractory cases or relapse prevention.^[20]^ Treatment outcomes across studies of skull base IgG4-RD generally favor corticosteroid therapy, with most patients showing substantial improvement. Marinelli et al reported that 91% of patients in the case series of skull base IgG4-RD experienced significant symptomatic response to therapy with steroids and rituximab, with 50% experiencing rapid response to corticosteroid alone before initiation of rituximab.^[3]^ Despite the therapeutic response, relapse is common in IgG4-RD, occurring in 24% to 54% of patients after reduction of corticosteroid dose.^[21,22]^ Thus, long-term follow-up of these patients is necessary.

## 4. Conclusion

Our case highlights central skull base osteomyelitis with deep neck abscesses as a novel clinical manifestation of IgG4-RD. IgG4-RD may not always manifest as a tumor-like mass de novo but may present with features of an infection, possibly related to immunosuppression from uncontrolled diabetes. In a patient with treatment-refractory skull base osteomyelitis and deep neck abscesses without an apparent cause, IgG4-RD should be considered as a differential diagnosis, and a tissue biopsy should be performed.

## 5. Patient perspective

The patient perspective, written in their own words in first person, provides a valuable insight into the lived experience of the disease. Unfortunately, we are not able to include this in our case report because we did not obtain a formal first-person account of the patient’s experience previously, and despite multiple attempts, we were unable to reestablish contact with the patient for this purpose.

## Acknowledgments

We thank the Director-General of Health Malaysia for permission to publish this article.

## Author contributions

**Conceptualization:** Shen-Han Lee, Nik Adilah Nik Othman.

**Resources:** Rohaizam Jaafar, Nurul Akmar Misron, Zulkifli Yusof.

**Supervision:** Nik Adilah Nik Othman.

**Visualization:** Shen-Han Lee.

**Writing – original draft:** Shen-Han Lee.

**Writing – review & editing:** Shen-Han Lee, Rohaizam Jaafar, Nurul Akmar Misron, Zulkifli Yusof, Nik Adilah Nik Othman.
